# Aligned Layers of Silver Nano-Fibers

**DOI:** 10.3390/ma5020239

**Published:** 2012-02-01

**Authors:** Andrii B. Golovin, Jeremy Stromer, Liubov Kreminska

**Affiliations:** 1 Department of Electrical Engineering, City College of the City University of New York, New York, NY 10031, USA; E-Mail: agolovin@ccny.cuny.edu; 2 Department of Physics & Physical Science, University of Nebraska at Kearney, Kearney, NE 68847, USA; E-Mail: stromerjd@lopers.unk.edu

**Keywords:** metal nano-fibers, lyotropic liquid crystals, aligned layers, dichroic polarizers, dichroic ratio, degree of polarization

## Abstract

We describe a new dichroic polarizers made by ordering silver nano-fibers to aligned layers. The aligned layers consist of nano-fibers and self-assembled molecular aggregates of lyotropic liquid crystals. Unidirectional alignment of the layers is achieved by means of mechanical shearing. Aligned layers of silver nano-fibers are partially transparent to a linearly polarized electromagnetic radiation. The unidirectional alignment and density of the silver nano-fibers determine degree of polarization of transmitted light. The aligned layers of silver nano-fibers might be used in optics, microwave applications, and organic electronics.

## 1. Introduction

Modern optical applications, e.g., flexible liquid crystal displays, require a polarizer with large optical aperture, minimized thickness, broad band transmission, and low cost. To resolve such contradictory requirements one may use so-called dichroic film polarizers. Historically the phenomenon of dichroism was first observed in mineral tourmaline by French physicist and mathematician Jean-Baptiste Biot in 1815. Tourmaline is semiprecious crystal boron silicate mineral which has a specific direction known as the principal or optic axis determined by its trigonal crystal structure. This mineral contains compounds of aluminum, iron, magnesium, sodium, lithium, or potassium. Tourmaline strongly absorbs light that is perpendicular to the optic axis of the crystal structure. A plate cut from tourmaline crystal parallel to its optic axis and several millimeters thick serve as a linear polarizer. The optic axis of tourmaline becomes the polarizer’s transmission axis. The intensity of transmitted light is wavelength dependent, and tourmaline plate viewed in natural white light is colored depending on mineral compounds. In the meantime, the same plate looks nearly black when viewed along the principal axis. These two colored appearances of the tourmaline gave the terms of dichroic crystal or dichroic polarizer.

The field of applications for dichroic polarizers made from natural tourmaline minerals is rather limited because the crystal size which is comparatively small. In contrast, in the majority of optical applications, the specially engineered dichroic film polarizers are used. The widely used dichroic type polarizers, so-called J-sheet and H-sheet, were invented by Edwin H. Land [1,2,3]. Both J- and H-sheets are made by using a syntactic dichroic substance called herapathite which was discovered by physician William B. Herapath and his pupil Mr. Phelps in 1852 by adding iodine into the urine of a dog that had been fed quinine [4]. Herapathite is a crystalline form of quinine sulfate per-iodide. To create J-sheet Land used needle-shaped the microscopic crystals of herapathite grinded in a ball mill for a month. The needle-shaped crystals were aligned nearly parallel to each other to form a layer of uniaxial crystal. To achieve parallel alignment, the herapathite needles suspended in a colloidal mixture were extracted through narrow slit. The commercial polarizer known as Polaroid J-sheet consists of oriented quinine herapathite needles in a cellulose acetate film. The J-sheets are first dichroic layers of created over large area of flat substrates. However, J-sheets demonstrate drawback by the light scattering on the individual microscopic needles of herapathite.

The H-sheet does not contain dichroic needle-shaped crystals, but it contains chains of polyiodide attached to the stretched polymer film of polyvinyl alcohol. Chains of polyiodide are formed along stretched hydrocarbon molecules of polymer, and are a molecular analogue of the grid of conductive wires. The electric field component of an incident wave that is parallel to the chains drives the electrons, does the work to induce a current through the chains, and is highly absorbed. Thus, the transmission axis of the polarizer is perpendicular to the direction of polyiodide chains. As advantages of H-sheet, one may mention an absence of light scattering and effective polarization of light across the entire visible spectrum. The H-sheets show slow process of a chemical decomposition accelerated by a high intensity of absorbed light. The H-sheet is a reliable polarizer at room temperature, but its performance is affected by high temperature since the iodine stain is desorbed at temperatures above 70 °C.

To improve the dichroic performance of H-sheet at elevated temperature Bloom used films of polyvinyl alcohol stained with metal chelates [5]. The molecularly oriented oligomeric metal chelates have the repeating structures, every unit of repeating structure contains metal molecules, e.g., nickel. The resultant polarizer exhibit excellent heat stability and is able to polarize light in wide spectral range, say from ultraviolet to infrared. The drawback of H-sheets with metal chelates is low concentration of metals within dichroic layers which gives the low polarization efficiency for one layer. To increase the efficiency, the dichroic layers might be stacked as multilayer coating in the path of light [6].

One may improve the dichroic performance of H-sheet by replacing the metal chelates with aligned nano-sized particles of noble metal such as silver nano-fibers. The nano-sized particles or just nano-particles should preserve the low level of light scattering because of their small dimensions, say, less than wavelength of radiation. The uniaxial alignment of silver nano-fibers would cause two plasmons: one with a strong longitudinal absorption of light linearly polarized parallel to the long axis of nano-fibers and other one with a weak absorption transverse to the long axis of nano-fibers [7]. The nano-fibers might be mono-dimensional to tune the resonant absorbance to a specific narrow wavelength range or they might be of poly-disperse to maintain a broad band absorbance. More importantly, the polarization performance of nano-fibers is free of aging decay. However, there are challenging questions about aligned layers of silver nano-fibers: How one may achieve a high concentration of silver nano-fibers and also align them into uniaxial structure of a thin dichroic layer?

Here we report about uniaxial layers of silver nano-fibers assembled by application a mechanical shearing to colloidal dispersion. Due to plasmon absorption, these uniaxial layers are capable of the dichroic absorption of visible and infrared radiation.

## 2. Results and Discussion

### 2.1. Overlapped Silver Nano-Fibers

**Figure 1:**
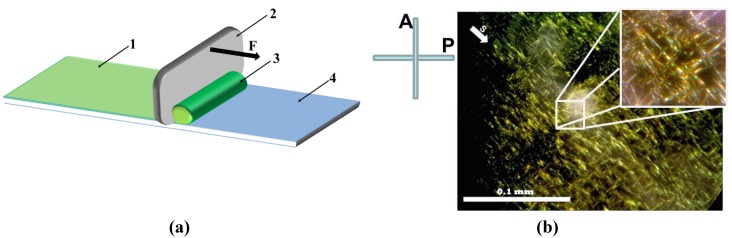
(Color online) (**a**) Shearing setup with the layer of silver nano-fibers (1), doctor blade (2) with applied force F, droplet of the colloidal dispersion (3), and bare borosilicate glass plate (4); (**b**) The microspore texture of Sample No. 1 with dried silver nano-fibers NGAP NF Ag-3101-W, which was captured between crossed polarizer (P) and analyzer (A). Vector S depicts the direction of shearing.

The metal nano-particles dispersed in coloidal solutions might be obtained by using the seed mediated growth method reported by B. Nikoobakht and M.A. El-Sayed [8]. This technology allows one to prepare metal nano-particles grown in micelles of cationic surfactants (e.g., hexadecyltrimethylammonium bromide (CTAB)). Molecules of CTAB create a bilayer of positively charged coat around each particle. On one hand, the long-range electrostatic repulsion of CTAB coats keeps nano-particles well dispersed in water. On other hand, the same repulsion presents difficulties for the uniaxial alignment of metal nano-particles. Figure 1 demonstrates an example of such alignment affected by repulsion between silver nano-fibers. To prepare this example of dried silver nano-fibers, a droplet of silver nano-fibers aqueous dispersion NGAP NF Ag-3101-W was sheared by using a metal doctor blade on the surface of bare borosilicate glass, Figure 1a. Once water evaporated, the silver nano-fibers were “frozen” in their positions. This dried layer of silver nano-fibers was marked as Sample No. 1. To observe the texture of frozen silver nano-fibers, Sample No. 1 was placed in view of polarizing microscope, between crossed polarizer P and analyzer A, Figure 1b. The silver nano-fibers are seen as long lines with mutual overlie angle of 90 degrees. Obviously one part of the nano-fibers was aligned along the shearing direction S and another part of nano-fibers is overlapped and oriented orthogonally to S direction. Such an orthogonal overlap texture indicates on the repulsive electrostatic interaction between the nano-fibers.

### 2.2. Silver Nano-Fibers Oriented by the Shearing Force

In 2008 Park and Lavrentovich [9,10] found technique for assembling gold nano-rods to either side-by-side or end-to-end stacks. Within this technique, the electrostatic interaction between nano-particles might be decreased by adding self-assembled molecular aggregates with electrical charges of the opposite sign into a common solvent such as water. The electrostatic attractive interaction acting between the nano-particles and molecular aggregates tie them together into assembled structures in water.

To prepare a dispersion of nano-particles stacks in nematic lyotropic liquid crystal we used the aqueous dispersion of silver nano-fibers NGAP NF Ag-3101-W and infrared dye IR806. It is known that the rod-shaped J-mesoaggregates of IR-806 dye form nematic lyotropic liquid crystals in aqueous solutions if its weight concentration is in the range 0.7–10% [11]. Thus, we added 0.0093 g of IR-806 to 1 g of NGAP NF Ag-3101-W to form 1% aqueous solution of infrared dye IR-806. After infrared dye was completely dissolved, a droplet of colloidal solution was sheared on the bare surface of borosilicate glass slide and left to dry at room temperature. After water evaporated this dried layer of nano-fibers was marked as Sample No. 2.

**Figure 2:**
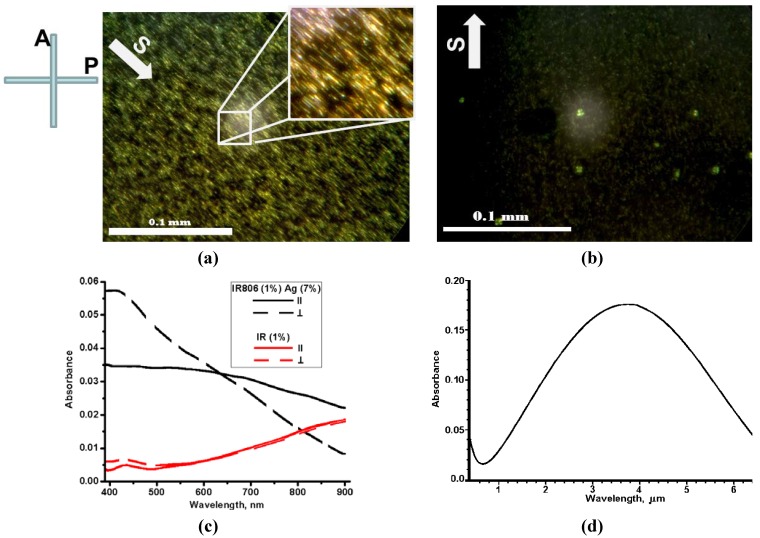
(Color online) (**a**) and (**b**) Textures of Sample No. 2 (dried silver nano-fibers with the infrared dye IR-806) placed between crossed polarizer P and analyzer A, with two different orientations of shearing direction S with respect to P and A; (**c**) Absorption spectra of Sample No. 2 (black line) and same for reference film of dried infrared dye IR-806 (red line) measured with visible and near-infrared lights linearly polarized along (∥) and orthogonally (⊥) to the shearing direction S; (**d**) Infrared absorption spectrum of the longitudinal plasmon calculated for the silver nano-fibers NGAP NF Ag-3101-W.

Figure 2 demonstrates textures of the dried Sample No. 2 placed between crossed polarizer P and analyzer A in the polarizing microscope. The dried silver nano-fibers were aligned along the shearing direction S. Also, texture in Figure 2a clearly demonstrates that the nano-fibers formed “raft” structures with side-by-side assemblies described by [9,10] for wet samples of gold nano-rods.

Absorption spectra of Sample No. 2 were measured with lights linearly polarized along (∥) and orthogonally (⊥) to the shearing direction S, Figure 2c. The linear polarization was achieved by using the pile of the quartz plates. Because lyotropic liquid crystals at certain concentrations and temperatures form aqueous nematic solutions, their sheared and then dried layers capable of the dichroic polarization (see for example References [12,13]), for the sake of comparison, Figure 2c demonstrates reference spectra of sheared dye IR-806 prepared in the similar way to Sample No. 2. The aligned silver nano-fibers in Sample No. 2 clearly demonstrate an anisotropic transmission of dichroic crystalline layer. In visible, from 400 to 650 nm, the high value of orthogonal absorbance (⊥) is caused by transvers plasmon absorption in the aligned nano-fibers. In further wavelength range, from 650 to 900 nm, the orthogonal absorbance decreased and longitudinal plasmon demonstrated dominance by increasing magnitude of the parallel absorbance (∥). By using simulations of optical absorption spectra described by M.A. El-Sayed [14], we modeled infrared absorption spectrum of the silver nano-fibers NGAP NF Ag-3101-W, Figure 2d. Maximum value of the parallel absorbance is anticipated in the center of longitudinal plasmon resonance at wavelength of 3.75 μm. Thus, the samples with aligned silver nano-fibers demonstrate even higher values of the parallel absorbance starting from near-infrared.

In order to increase the load of silver nano-fibers, the solution NGAP NF Ag-3101-W was centrifuged for 30 minutes with frequency of rotation 8,000 rpm. Amount of deionized water was decreased, and then the silver sediment was re-dispersed by using an ultrasound bath. The weight concentration of the silver nano-fibers in water was increased up to 35%.

Then, in order to preserve the weight concentration of the infrared dye at the level of 1%, 0.0066 g of IR-806 dye were added to 1 g of the concentrated aqueous dispersion of silver nano-fibers. After the infrared dye was completely dissolved, solution was sheared on the glass substrate similarly to the previous two samples. New sample was left to dry at room temperature and marked as Sample No. 3.

**Figure 3:**
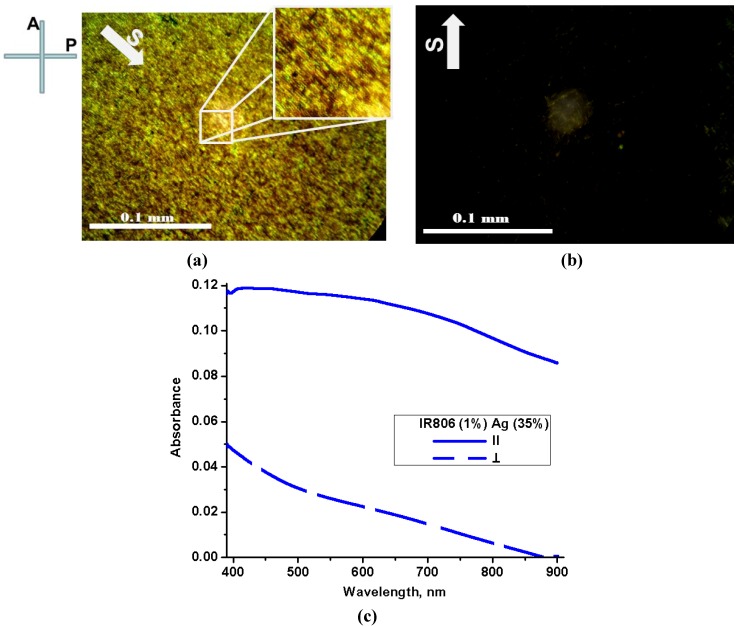
(Color online) (**a**) and (**b**) Textures of Sample No. 3 (concentrated silver nano-fibers with the infrared dye IE-806) placed between crossed polarizer P and analyzer A, with two different orientations of shearing direction S with respect to P and A; (**c**) Absorption spectra of Samples 3, (blue lines) measured with lights linearly polarized along (∥) and orthogonally (⊥) to the shearing direction S.

Figure 3a,b demonstrates textures of the dried Sample No. 3 placed between crossed polarizer P and analyzer A in the polarizing microscope. The dried silver nano-fibers were aligned in uniaxial fashion along the shearing direction S, Figure 3a.

Absorption spectra of Sample No. 3 were measured in linearly polarized lights as absorption spectra of Sample No. 2 and depicted in Figure 3c.

**Figure 4:**
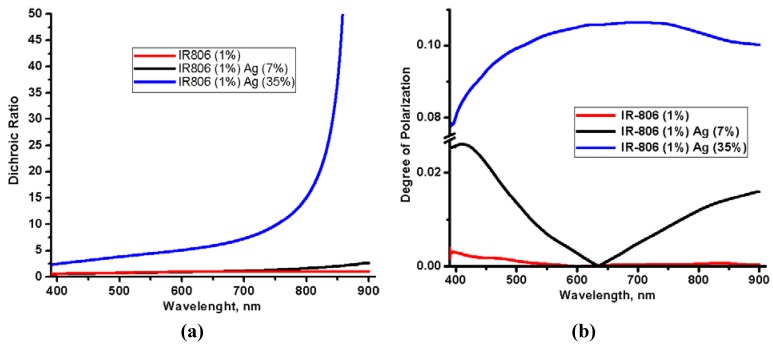
(Color online) (**a**) Dichroic ratio and (**b**) degree of polarization of reference layer of IR-806 (red), Samples No. 2 (black), and Samples No. 3 (blue).

The higher load of silver nano-fibers improves dichroic properties of the aligned structure of silver nano-fibers. Figure 4a demonstrates such improvement of dichroic properties by depicting spectra of the dichroic ratios *R* defined as *R = A_∥_ ⁄A_⊥_*, were *A_∥_* and *A_⊥_* are absorbance measured with lights linearly polarized along (∥) and orthogonally (⊥) to the shearing direction S, respectively. The dichroic ratio of the layer with the low load of silver nano-fibers (Sample No. 2) demonstrates minor improvement over the dichroic ratio of the reference layer with the infrared dye IR-806 (compare black and red lines). The layer with the high load of the silver nano-fibers (Sample No. 3) demonstrates at least one order higher value of dichroic ratio (blue line) in comparison with other two spectra. The improvement is much higher in the near infrared range, which is closer to center of the longitudinal plasmon absorption of aligned silver nano-fibers.

Figure 4 also describes improvement of linear polarization of light transmitted by the Samples No. 3. We used concept of the degree of polarization [15,16] defined as a ratio *V = (I_max_ − I_min_)/(I_max_ + I_min_)*, where *I_min_* and *I_max_* are maximum and minimum intensities of linearly polarized light transmitted along and orthogonally to the shearing direction S, respectively. Figure 4b demonstrates that the degree of polarization of light after Sample No. 3 is higher by one order of magnitude than degrees of polarization of Sample No. 2 and reference layer of IR-806 dye.

## 3. Experimental Section

With regards to the following description of experiments, the aqueous solution of colloidal silver nano-fibers NGAP NF Ag-3101-W (with average length of 20 microns and diameter of 100 nanometers, Zeta potential *ζ* ≈ −20 mV, weight concentration in water of 7% (data supplied by manufacturer) were purchased from NANOGAP, Spain; the infrared dye IR-806 was ordered from Sigma-Aldrich and used without further purification. The samples of dried dichroic layers were prepared on the borosilicate glass slides from Sailing Boat, China. The textures were photographed by using a polarizing microscope ML9000 with attached CCD camera Infinity 1 from Meji Techno CO. LTD, Japan. Absorption spectra of samples were measured by using UV-Vis spectrometer Lambda 14 from Perkin-Elmer.

## 4. Conclusions

We have prepared colloidal dispersions with side-by-side assemblies of silver nano-fibers linked by self-organized lyotropic cromonic materials. We have prepared uniaxial layers of dried silver nano-fibers aligned by using mechanical shearing process of the colloidal dispersions on the bare surfaces of glass substrates. These uniaxial layers are capable of linear polarization as dichroic polarizers for visible and infrared radiation. In the infrared part of spectrum the layers demonstrate a nonlinear increase of dichroic ratio caused by longitudinal plasmon absorption of aligned silver nano-fibers.
